# Family and case–control genetic study of *MSX1* polymorphisms in peg-shaped teeth Jordanian population

**DOI:** 10.1186/s12903-022-02051-2

**Published:** 2022-01-22

**Authors:** Rami Alkhatib, Razan Hawamdeh, Laith Al-Eitan, Nour Abdo, Fadi Obeidat, Mohamed Al-Bataineh, Hatem Aman

**Affiliations:** 1 Department of Applied Biological Sciences, Jordan University of Science and Technology, Irbid, 22110 Jordan; 2 Department of Biotechnology and Genetic Engineering, Jordan University of Science and Technology, Irbid, 22110 Jordan; 3 Department of Public Health, Faculty of Medicine, Jordan University of Science and Technology, Irbid, 22110 Jordan; 4 Department of Dentistry, Jordanian Royal Medical Services, Irbid, Jordan

**Keywords:** *MSX1*, Allele frequency, SNPs, Exons, Primers

## Abstract

**Background:**

This study aimed to investigate the genetic association of specific Single Nucleotide Polymorphisms (SNPs) within the muscle segment homeobox gene 1 (*MSX1*) with susceptibility to the peg-shaped teeth in 36 Jordanian Arab families and case–control samples in the Jordanian Arab population.

**Methods:**

This cohort involved 108 individuals (36 trios families), which were used for family-based genetic study. Additionally, 56 patients and 57 controls were used for case–control study. Genomic DNA samples from both families and case–control were extracted according to distinguished processes. Then, polymerase chain reaction technique (PCR) was conducted using specific primers for the axons of the *MSX1*. Moreover, DNA sequencing genotyping method analysis of SNPs was used to detect specified SNPs in the *MSX1* linked with peg-shaped teeth. Hardy–Weinberg Equilibrium and *Chi*-square were used to evaluate the data quality and the presence of any genotypic error. In addition, Transmission Disequilibrium Test (TDT) was used identify family-based association in which trios of parents and proband are used.

**Results:**

The results of this study showed fourteen polymorphic sites in this gene, eight of them (rs121913129, rs104893852, rs104893853, rs121913130, rs104893850, rs1095, rs3775261, and rs1042484) were none-polymorphic. Meanwhile, the minor allele frequencies of the rest of the SNPs were polymorphic (rs8670, rs12532, rs3821949, rs4464513, rs1907998, and rs6446693). However, none of these SNPs were associated with peg-shaped teeth. Moreover, the haplotype genetic analysis revealed that there was no genetic association with peg-shaped teeth disorder susceptibility (*P* > 0.05) in the Jordanian families of Arab descent.

**Conclusions:**

The present findings can be used in estimation of prevalence of peg-shaped teeth in the Jordanian population. However, our findings revealed that there is no evidence that the *MSX1* polymorphisms had a crucial role in the peg-shaped teeth phenomenon, emphasizing that other genes might have this role. These findings are beneficial for clinicians to comprehensively understand the molecular aspects of teeth abnormalities.

**Supplementary Information:**

The online version contains supplementary material available at 10.1186/s12903-022-02051-2.

## Background

Tooth agenesis is among the well-recognized morphological anomalies in humans. Peg-shaped teeth are a hereditary dental disorder called microdontia, a condition where one or more teeth appear smaller than average (microdontia) [1]. In general, the most common teeth affected are the upper lateral incisors or sometimes third molars. Moreover, it might be seen on both sides in most instances, and they have shorter roots than usual teeth [2]. Peg-shaped teeth are characterized by autosomal dominant inheritance that might cause defects during teeth development. As a result, there is an insufficient development compared to normal development. The prevalence of agenesis in the permanent dentition, excluding the third molars, ranges between 0.15 and 16.2% [3] with a higher prevalence in females than males [4–6]. Hua et al. [7] reported that women were 1.35 times more likely than men to have peg-shaped maxillary permanent upper lateral incisors. In general, the prevalence of peg-shaped teeth is about 1.8% [7]. The prevalence of tooth agenesis in permanent teeth in both genders varies among different populations. For example, in Europe the prevalence is 4.6% in males and 6.3% in females; in North American Caucasians is 3.2% in males and 4.6% in females. Meanwhile, the prevalence was the highest in Australia (males 5.5% and females 7.6%) [8].

Odentogenesis undergoes restricted genetic and morphological manipulation depending on cell–cell interactions resulting in the initiation and generation of tooth induced by morphological signaling pathway [9, 10]. Thus, any defect in the germ tooth can lead to dental anomalies either in shape, number, structure, or size of the teeth which exhibit morphological anomalies in humans [11]. Numerous genes and signaling pathways participate in tooth formation and cell differentiation at specific stages of odentogenesis [12] which may cause mutation in tooth agenesis [13]. Dental anomalies could be a combination between genetic, epigenetic and environmental factors during the process of dental development [14]. There are many genes involved in peg-shaped teeth. More than 350 genes have been associated with teeth development such as *PAX9*, *MSX1*, *AXIN2*, *EDA*, *EDAR and WNT10a* [15]. However, few genetic studies reported how genes are related to this disorder. In Jordanian population, the effect of *MSX1* variation on the peg-shaped teeth is still not clear. The rate of persons with peg-shaped teeth disorder has been increased with a lack of information about the causes of this disorder and occurrence. According to several studies on this disorder, various environmental factors have been investigated in details with very restricted attention has been paved to the genetic component factor [1]. Therefore, a multi genetic factorial epidemiology study has been actually focusing on the genetic aspects including several genes such as *MSX1* [16].

Expression assays and transgenic mouse phenotypes revealed that *MSX1* has a critical role in craniofacial development [17]. The *MSX1* is expressed in the mesenchyme of developing tooth germ especially at the bud and cap stages as a response to epithelial signals [18]. Muscle segment homebox is a non-clustered home box protein, which is located in the small arm of chromosome 4 (chromosome 4p16.2) [19]. It contains two exons which are separated by an intron. The *MSX1* is a member of the mammalian *MSX* gene family, which consists of three physically unlinked members (*MSX1*, *MSX2*, and *MSX3*). *MSX* genes are essential for normal craniofacial, limb and ectodermal organ morphogenesis, and are also essential to survival in mice [20]. The MSX1 is a protein that in humans is encoded by *MSX1* [21]. The encoded protein acts as transcription factors repressor during morphogenesis through interaction with components of the core transcription component complex and other home proteins. Phenomena caused by the lack of *MSX1* protein may depend on the localization of the mutations and their effect on the protein structure and function. Moreover, a polymorphism in the *MSX1* leads to hypodontia and most of the affected teeth were second premolar and third molar. In addition, the upper lateral incisor, upper first molar, lower central incisor, and lower first molar could be absent [17, 22]. For example, mice with a homozygous deletion of *MSX1*exhibit a complete cleft palate and failure of tooth development [23].

The objective of this study was to investigate the genetic association of the *MSX1* and its susceptibility to the peg-shaped teeth in 36 families and case–control samples Jordanian Arab population.

## Methods

### Participants

Ethical approval for this study was in compliance with the Institutional Review Board (IRB) Guidelines at Jordan University of Science and Technology (IRB# 19/85/2015). All clinical investigations were conducted according to the principles expressed in the Declaration of Helsinki consent. Participants in this study consisted of 36 Jordanian Arab families with peg-shaped teeth cases and their biological parents; 137 samples were involved in this study (58 males [42.3%] and 79 females [57.7%]). The 137 samples consisted of 36 trios (108 individuals) and additional 29 unrelated individual cases. Of the latter 29 cases, nine cases failed genotyping and were excluded in the final case–control analyses. A total of 57 controls were added to the case–control study (Fig. S1). Peg-shaped teeth were diagnosed by a specialized dentist based on the morphology of the tooth only (cone shape). All cases of peg-shaped teeth used in this study were permanent upper lateral incisors only. Clinical and demographic data from each participant were collected using a semi-structured interview, which was built on a standard protocol. Demographic data including gender and age were collected (Table 1). Written informed consent was provided by all subjects in this study.

**Table 1:** Characteristics of 36 Peg-shaped teeth families of Jordanian descent in this study

Characteristics of patients	N (%)
Age (years)	
9–29	6 (48.2)
30–50	50 (36.5)
51–71	18 (13.1)
72–92	3 (2.2)
Gender	
Male	58 (42.3)
Female	79 (57.7)
Position of peg-shaped tooth	
Bilateral	26 (19.0)
Upper right	14 (10.2)
Upper left	12 (8.8)
Other	85 (62.0)
Other anomalies	
Hyperdontia (> 32 teeth in the mouth)	1 (0.7)
Hypodontia (1–6 teeth excluding third molar)	12 (8.8)
Macrodontia (1 or 2 teeth larger than normal)	3 (2.2)
Microdontia (1 or 2 teeth smaller than normal)	5 (3.6)

### The candidate genes and the selected SNPs

In this study, National Centre for Biotechnology Information (NCBI), SNPs Database (dbSNP), and HapMap project databases were used to select the SNPs, which were selected based on: Map position, minor allele frequency, and previous SNPs studies. The *MSX1* and its SNPs and positions, and genotyping data based on the whole cohort (137 Subject) is shown in (Table 2).The rational for selecting these SNPs was: SNPs with significant functional relevance, SNPs that can cover the genes of interest as widely as possible, and SNPs that had been already genotyped in other studies. Moreover, these SNPs had a higher ability to be implicated in several tooth disorders and may have a major effect on peg-shaped teeth cases. Based on that, fourteen SNPs in chromosome 4 were selected.

**Table 2:** The basic information of the selected SNPs in this study

SNP ID	Position^a^	SNP	SNP location
rs121913129	4862836	C>G	MISSENSE
rs104893852	4860231	A>C	STOP GAIND
rs104893853	4862854	A>C	STOP GAIND
rs121913130	4860099	A>T	MISSENSE
rs104893850	4862808	T>C	STOP GAIND
rs8670	4863149	C>T	UTR variant 3 prime
rs1095	4863211	C>T	UTR variant 3 prime
rs12532	4863419	A>G	UTR variant 3 prime
rs3821949	4858675	A>G	Upstream variant 2 KB
rs4464513	4865595	G>T	By 1000G, by 2hit 2allele, by cluster, by frequency
rs3775261	4862018	A>G	Intron variant
rs1042484	4862654	C>T	Intron variant
rs1907998	4854852	A>G	By 1000G,by 2hit 2allele, by cluster, by frequency, by HapMap, by submitte
rs6446693	4853353	C>T	By 1000G,by 2hit 2allele, by cluster, by frequency, by HapMap

^a^Chromosome positions are based on NCBI Human Genome Assembly Build 4p16.2

### DNA collection and analysis

DNA was extracted using commercially available kit (Promega, USA) according to the manufacturer’s instructions. Then, the DNA samples were diluted making the DNA concentration to be 20 ng/μl. DNA quantity and purity were verified using Nano-DropND-1000 UV-V Spectrophotometer (Bio-Drop Spectrophotometer, Cambridge, UK).

### DNA genotyping

DNA samples were genotyped using the Sequenom Mass ARRAY® system (IPLEX GOLD) (Sequenom, San Diego, CA, USA) in the Australian Genome Research Facility [AGRF] (Melbourne Node, Australia).

### Statistical and haplotype analyses

Hardy–Weinberg Equilibrium (HWE), Mendelian inheritance of the genotypes, and haplotype test were used in this study. Hardy–Weinberg Equilibrium and *Chi*-square were used to evaluate the data quality and the presence of any genotypic error. The genetic differences in allele frequencies and genotype distribution of each polymorphism of interest in the family study and case–control study were compared using the *Chi-square* test. Moreover, odd ratio was calculated with 95% confidence interval (CI). In addition, haplotype test was used for Linkage Disequilibrium (LD) for each individual polymorphism using Haploview Software (Version 4.2). Transmission Disequilibrium Test (TDT) was used identify family-based association in which trios of parents and proband are used. Stratified genotype analysis for each SNP was calculated using PC SAS (v. 9.2; SAS Institute, Cary, NC, USA).

## Results

### Hardy–Weinberg equilibrium test (HWE)

All the genotyped polymorphisms met the HWE and Mendelian errors tests for the case–control and family groups. The Sequenom MassARRAY® system data was very precise with an average success rate of 99%. No Mendelian errors were observed. The 14 SNPs in *MSX1* gene (Chromosome 4q16.2) were used; eight of them (rs121913129, rs104893852, rs104893853, rs121913130, rs104893850, rs1095, rs3775261, and rs1042484) were none-polymorphic. Meanwhile, the minor allele frequency of the other six SNPs was polymorphic (rs8670, rs12532, rs3821949, rs4464513, rs1907998, and rs6446693). The minor alleles of the studied SNPs and their frequencies for both families and case–control are shown in (Table 3).

**Table 3:** The *MSX1* SNPs with their minor allele frequencies and HWE *P*-values for cases and controls at each locus based on the (36) families

Gene	SNP ID	(36) families			Controls n = (57)			Cases n = (56)		
		MA	MAF	HWE*P*-value	MA	MAF	HWE*P*-value	MA	MAF	HWE*P*-value
*MSX1*	rs12532	G	0.28	0.077	G	0.31	0.270	G	0.04	0.000
	rs1907998	G	0.35	0.721	G	0.24	0.741	G	0.36	0.434
	rs3821949	A	0.23	0.934	A	0.27	0.040	A	0.22	0.688
	rs4464513	T	0.44	0.942	T	0.36	0.934	T	0.39	0.926
	rs6446693	C	0.47	0.001	C	0.46	0.742	C	0.45	0.112
	rs8670	T	0.23	0.643	T	0.27	0.536	T	0.29	0.309

*MA* minor allele, *MAF* minor allele frequency, *HWE* Hardy–Weinberg equilibrium

### Association of SNPs candidate genes with peg-shaped teeth

**Fig. 1:**
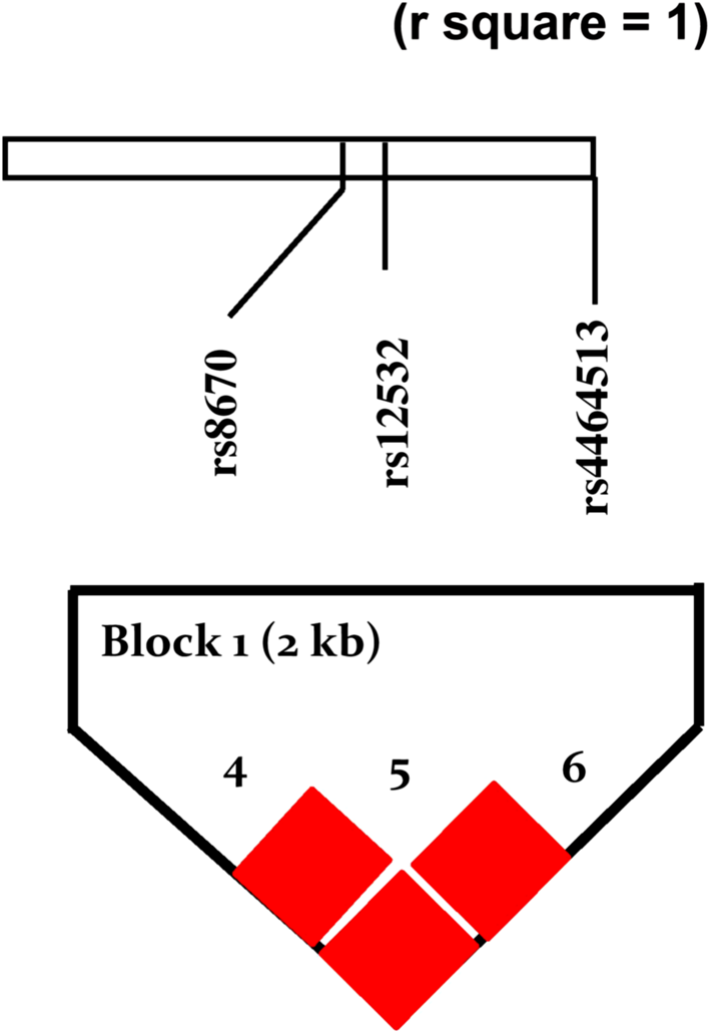
Haploview plot of linkage disequilibrium (r^2^) between (rs8670, rs12532, and rs4464513) within *MSX1* located on chromosome 4p16.2 represent SNP-SNP relationship. A black diamond without a number represents complete linkage disequilibrium between SNPs (r^2^ = 1)

The distribution of alleles and genotypes of the genotyped SNPs in the peg-shaped teeth patients and their parents, brothers, and sisters were recorded. In addition, the association of these SNPs was tested using the TDT. The TDT test was carried out on 36 trios (male and female probands) and exhibited no significant preferential transmission in any of the six SNPs studies (Table 4). The observed value of D, a representation of allelic frequencies of SNPs, was not equal to 1, suggesting that the SNPs were in perfect LD. The genetic haplotype block of rs8670, rs12532, and rs4464513 within the *MSX1* was shown in (Fig. 1). The haplotype program revealed that one SNP (rs6446693) out of the six SNPs from Sequenom Massarray data was not associated with peg-shaped teeth.

**Table 4:** The Transmission Disequilibrium Test (TDT) analysis for allelic association in 36 Peg-shaped tooth trios in Jordanian Population

SNP ID	Allele	T^a^	NT^b^	Ratio (T/NT)	*X*^2^	*P-*value^c^
rs12532	A	G	A	12:70	1.316	0.251
	G					
rs1907998	A	A	G	14:12	0.154	0.695
	G					
rs3821949	A	A	G	12:11	0.043	0.835
	G					
rs4464513	T	G	T	14:60	3.200	0.074
	G					
rs6446693	T	T	C	20:16	0.444	0.505
	C					
rs8670	T	T	C	12:90	0.429	0.513
	C					

^a^Transmitted allele

^b^None transmitted allele

^c^*P*-value is significant when *P* < 0.05

**Fig. 2:**
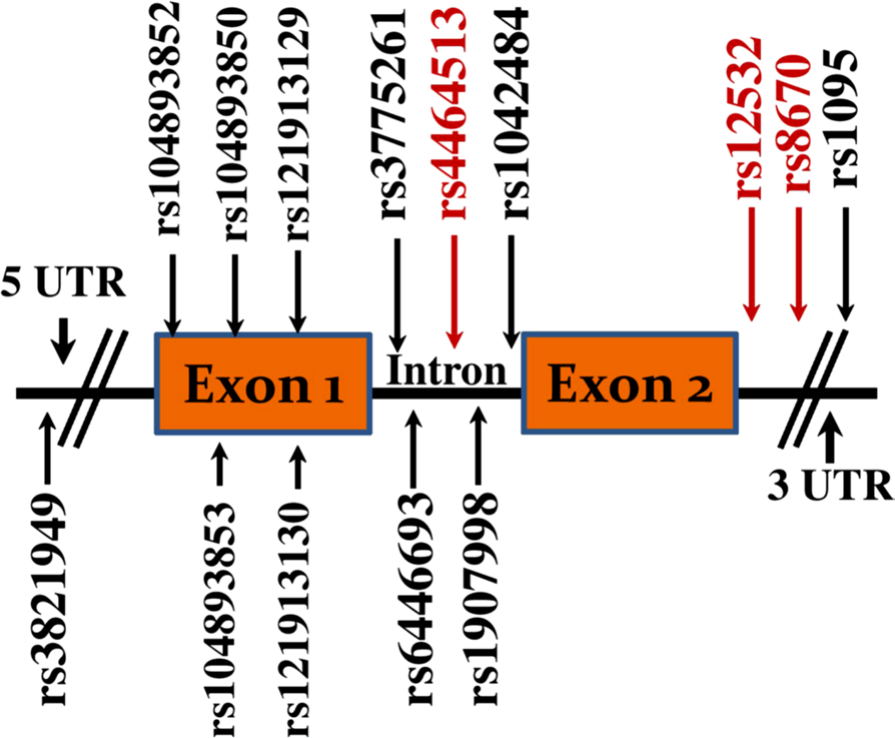
Schematic structure of the human *MSX1* showing the positions of the SNPs genotyped and their dbSNP IDs

The genetic association analysis of all six SNPs in peg-shaped teeth cases and controls using different genetic models are exhibited (Table S1). The statistical analysis for the heterozygous versus both common homozygous and rare homozygous or even testing the latter two together was performed (Table S2). Moreover, stratified genotype analysis by gender for each SNP (adjusted by age) was conducted (Table 5). For case and control samples, all genotype SNPs were checked for HWE and Mendelian errors. The minor allele frequency for all six SNPs was within normal distribution in both studies (*P* = 0.05). The genotype frequencies of all six SNPs (rs12532, rs1907998, rs3821949, rs4464513, rs6446693, and rs8670) within the *MSX1* displayed no association with peg-shaped teeth in both studies. Schematic structure of the human *MSX1*, and the positions of the fourteen SNPs genotyped and their db SNP IDs displayed in Fig. 2.

**Table 5:** Stratified genotype analysis for each SNP (by gender and adjusted by age)

SNP	Genotype	Gender	Cases	Controls	Odds ratio (95% CI)	P-value
rs2073244	A/A	Female	18	5	1.00	0.039
		Male	2	4	12.14 (1.13–130.56)	
	G/A	Female	14	12	1.00	0.887
		Male	7	5	0.89 (0.20–3.92)	
	G/G	Female	6	2	1.00	0.897
		Male	4	1	1.24 (0.06–25.14)	
rs2073246	C/C	Female	18	5	1.00	0.039
		Male	2	4	11.66 (1.12–121.43)	
	C/T	Female	15	12	1.00	0.802
		Male	7	4	0.81 (0.17–3.76)	
	T/T	Female	5	2	1.00	0.946
		Male	4	1	0.89 (0.04–18.48)	
rs2295222	C/C	Female	22	7	1	0.157
		Male	5	4	3.59 (0.61–21.04)	
	C/A	Female	15	12	1.00	0.982
		Male	7	4	0.98 (0.20–4.73)	
	A/A	Female	3	0	1.00	
		Male	1	1	–	
rs4904155	C/C	Female	16	5	1.00	0.059
		Male	2	4	9.82 (0.91–105.47)	
	G/C	Female	14	12	1.00	0.664
		Male	7	4	0.70 (0.15–3.30)	
	G/G	Female	8	2	1.00	0.703
		Male	4	1	1.84 (0.09–35.81)	
rs4904210	G/G	Female	16	4	1.00	0.085
		Male	2	3	8.70 (0.74–102.10)	
	G/C	Female	16	12	1.00	0.787
		Male	6	6	1.23 (0.30–5.10)	
	C/C	Female	6	2	1.00	0.871
		Male	4	1	1.30 (0.07–25.13)	

*CI* confidence interval

## Discussion

To the best of our knowledge, this is the first study that has analyzed the association of *MSX1* in peg-shaped teeth syndrome in the Jordanian population of Arab descent. In addition, these individuals were characterized by typical symptoms such as tooth phenotypic structure that look like tapered maxillary lateral incisor which is commonly called morphological anomalies conical shape (The emergence of sharp teeth clearly like a screw) [24].

In this study, the sample set included 36 families with at least one affected child with peg-shaped teeth syndrome. The results of the current study revealed no significant association of the fourteen SNPs with the peg-shaped teeth in all samples in both genders. Even though, it has been reported that inactivation of *MSX1* and *PAX9* in mice showed that these genes are relevant to dental and craniofacial development [23, 25]. Interestingly, Alkhatib et al. (2020) reported that two SNPs in the paired box gene 9 (*PAX9*) were significantly associated with peg-shaped teeth in the same subjects used for this current study [26]. In this study, the 36 families were separated according to the gender of patients (male-only (MO) comparing with female-containing (FC) families) and analyzed based on a specific gender. However, the results showed that there was no significant association of these SNPs with the peg-shaped teeth in families with affected males only or in families with affected females only in trios [27].

Numerous former researches indicated that there is a genetic linkage in the small arm of chromosome 4 (4p16.2). Moreover, there is strong evidence that *MSX1* is expressed in the developing craniofacial region [19, 23]. Based on their findings, there are no investigated results or evidences that confirm a significant genetic association between the *MSX1* and peg-shaped teeth, which is similar to the presented results in this study. Alvesalo and Portin reported that agenesis and peg-shaped teeth could be autosomal dominant genes with reduced penetrance [28]. This reinforces our conclusion in the present study that there is an ethnic marker due to a genetic heterogeneity of the studied population in different ethnicities. The reason for these conflicting results might be firstly due to the ethnic differences between populations which contribute to genetic variation. Secondly, this study included only the Jordanian population of Arab ethnicity, which is considered as an isolated population. Thirdly, the genetic heterogeneity of peg-shaped teeth disorder and the possibility of the involvement of other genes or environmental factors such as gene–gene interaction or gene-environmental interaction must be considered for further research. This is also the limitation of this study.

Finally, there was a lack of information about environmental risk factors. Also, the promoter of this gene which has a direct effect on the regulation of the *MSX1* expression was not investigated in this study.

## Conclusion

This is the first genetic project of the *MSX1* in the Jordanian peg-shaped teeth patients. No prior studies have been carried out investigating the *MSX1* and its association with the peg-shaped teeth in the Arab population. These results revealed that there was no evidence that the *MSX1* polymorphisms play a crucial role in peg-shaped teeth phenomenon. More genetic studies on Jordanian peg-shaped teeth in Arab population should be explored in the future.

## Supplementary Information

**Additional file 1. Table S1**: Genetic Models Analyses for each SNP adjusted by gender and age.

**Additional file 2. Table S2**: Genetic association analysis of all six SNPs polymorphisms in peg-shaped tooth cases and controls using different genetic models.

**Additional file 3. Fig. S1**: Genetic association analysis of all six SNPs polymorphisms in peg-shaped tooth cases and controls using different genetic models.

## Data Availability

All data generated or analysed during this study are included in this published article. All relevant raw data will be freely available to any researcher wishing to use them for non-commercial purposes, without breaching participant confidentiality using the following link below: (https://mega.nz/file/bHoSnbhZ#oLWIgrBxe6UCRsZUn4ZlBeGrhfyln2Ep_3u8X61dWSM).

## Abbreviations

AGRF: Australian Genome Research Facility
DNA: Deoxyribonucleic acid
dbSNP: Single Nucleotide Polymorphism Database
HWE: Hardy–Weinberg equilibrium
IRB: Intuitional Review Board
LD: Linkage disequilibrium
NCBI: National Centre for Biotechnology Information
PCR: Polymerase chain reaction
SNP: Single nucleotide polymorphism
*MSX1*: Muscle Segment Homeobox Gene 1
MAFs: Minor allele frequencies
TDT: Transmission Disequilibrium Test

## References

[1] Devasya A, Sarpangala M (2016). Dracula tooth: a very rare case report of peg-shaped mandibular incisors. J Forensic Dent Sci.

[2] Abusalih A, Ismail H, Abdulgani A, Chlorokostas G, Abu-Hussein M (2016). Interdisciplinary management of congenitally agenesis maxillary lateral incisors: orthodontic/prosthodontics perspectives. J Dent Med Sci.

[3] Rakhshan V (2015). Congenitally missing teeth (hypodontia): a review of the literature concerning the etiology, prevalence, risk factors, patterns and treatment. Dent Res J.

[4] Khalaf K, Miskelly J, Voge E, Macfarlane TV (2014). Prevalence of hypodontia and associated factors: a systematic review and meta-analysis. J Orthod.

[5] Laganà G, Lombardi CC, Franchi L, Cozza P (2011). Tooth agenesis: dento-skeletal characteristics in subjects with orthodontic treatment need. Eur J Paediatr Dent.

[6] Laganà G, Masucci C, Fabi F, Bollero P, Cozza P (2013). Prevalence of malocclusions, oral habits and orthodontic treatment need in a 7- to 15-year-old schoolchildren population in Tirana. Prog Orthod.

[7] Hua F, He H, Ngan P, Bouzid W (2013). Prevalence of Peg-shaped maxillary permanent lateral incisors: a meta-analysis. Am J Orthod Dentofac Orthop.

[8] Polder BJ, Van’t Hof MA, Van der FP, Kuijpers‐Jagtman AM. A meta‐analysis of the prevalence of dental agenesis of permanent teeth. Community Dent Oral Epidemiol. 2004;32(3):217–26. 10.1111/j.1600-0528.2004.00158.x. 10.1111/j.1600-0528.2004.00158.x 15151692

[9] Cobourne MT. The genetic control of early odentogenesis. Brit J Orthodont. 1999;26(1):21–8. 10.1093/ortho/26.1.21. 10.1093/ortho/26.1.21 10333884

[10] Bhuvan N, Usha H, Archana S (2016). Recent concepts of odentogenesis with applied aspects.

[11] Jahanimoghadam F (2016). Dental anomalies: an update. Adv Hum Biol.

[12] Pispa J, Thesleff I (2003). Mechanisms of ectodermal organogenesis. Dev Biol.

[13] Thesleff I (2000). Genetic basis of tooth development and dental defects. Acta Odontol Scand.

[14] Laganà G, Venza N, Borzabadi-Farahani A, Fabi F, DanesiC CP (2017). Dental anomalies: prevalence and associations between them in a large sample of non-orthodontic subjects, a cross-sectional study. BMC Oral Health.

[15] Bonczek O, Bielik P, Krejčí P, Zeman T, Izakovičová-Hollá L, Šoukalová J, Vaněk J, Gerguri T, Balcar VJ, Šerý O (2018). Next generation sequencing reveals a novel nonsense mutation in *MSX1* gene related to oligodontia. PLoS ONE.

[16] Watted N, Abu-Hussein M (2016). Multidisciplinary aesthetic dental treatment; peg lateral with congenitally maxillary lateral incisors. J Dent Med Sci.

[17] Lidral AC, Reising BC (2002). The role of *MSX1* in human tooth agenesis. J Dent Res.

[18] Abu-Hussein M, Watted N, Hegedűs V, Borbély P, Azzaldeen A (2015). Human genetic factors in non-syndromic cleft lip and palate: an update. Int J Maxillofac Res.

[19] Alappat S, Zhang ZY, Chen YP (2003). MSX1 homeobox gene family and craniofacial development. Cell Res.

[20] Hewitt JE, Clarka LN, Ivens A, Williamsona R (1991). Structure and sequence of the human homeobox gene HOX7. Genomics.

[21] Blin-Wakkach C, Lezot F, Ghoul-Mazgar S, Hotton D, Monteiro S, Teillaud C, Pibouin L, Orestes-Cardoso S, Papagerakis P, Macdougall M, Robert B, Berdal A (2001). Endogenous MSX1 antisense transcript: in vivo and in vitro evidences, structure, and potential involvement in skeleton development in mammals. Proc Natl Acad Sci.

[22] Vastardis H (2000). The genetics of human tooth agenesis new discoveries for understanding dental anomalies. Am J Orthodont Dentofac Orthoped.

[23] Satokata I, Maas R (1994). MSX1 deficient mice exhibit cleft palate and abnormalities of craniofacial and tooth development. Nat Genet.

[24] Kondo S, Townsend G, Matsuno M (2014). Morphological variation of the maxillary lateral incisor. Jpn Dent Sci Rev.

[25] Neubuser A, Peters H, Balling R, Martin GR (1997). Antagonistic interactions between FGF and BMP signaling pathways: a mechanism for positioning the sites of tooth formation. Cell.

[26] Alkhatib R, Obeidat B, Al-Eitan L, Abdo N, Obeidat F, Aman H (2020). Family-based association study of genetic analysis of paired box gene 9 polymorphisms in the peg-shaped teeth in the Jordanian Arab population. Arch Oral Biol.

[27] Bronckers AL, Lyaruu DM, DenBesten PK. The impact of fluoride on ameloblasts and the mechanisms of enamel fluorosis. J Dent Res. 2009;88(10):877–93. 10.1177/0022034509343280. 10.1177/0022034509343280 PMC3318083 19783795

[28] Alvesalo L, Portin P (1969). The inheritance pattern of missing, peg-shaped, and strongly mesio-distally reduced upper lateral incisors. Acta Odontol Scand.

