# Supplementary material for: Family and case–control genetic study of MSX1 polymorphisms in peg-shaped teeth Jordanian population
Source: BMC Oral Health. 2022 Jan 22;22:16. doi: 10.1186/s12903-022-02051-2 (PMC8783454; doi:10.1186/s12903-022-02051-2)
Supplement: Supplementary file 1 — Additional file 1. Table S1: Genetic Models Analyses for each SNP adjusted by gender and age. [file 12903_2022_2051_MOESM1_ESM.docx]

**Table S1.** Genetic Models Analyses for each SNP adjusted by gender and age.

| **SNP** | **Model** | **Genotype** | **N (%)** | | **OR (95% CI)^a^** | **P-value** | **AIC^b^** | **BIC^c^** |
| --- | --- | --- | --- | --- | --- | --- | --- | --- |
|  |  |  | **Cases** | **Controls** |  |  |  |  |
| **rs2073244** | **Codominant** | A/A | 20 (39.2%) | 9 (31%) | 1 | 0.58 | 101 | 112.9 |
|  |  | G/A | 21 (41.2%) | 17 (58.6%) | 1.36 (0.45-4.04) |  |  |  |
|  |  | G/G | 10 (19.6%) | 3 (10.3%) | 0.63 (0.12-3.23) |  |  |  |
|  | **Dominant** | A/A | 20 (39.2%) | 9 (31%) | 1 | 0.78 | 100 | 109.6 |
|  |  | G/A-G/G | 31 (60.8%) | 20 (69%) | 1.16 (0.40-3.31) |  |  |  |
|  | **Recessive** | A/A-G/A | 41 (80.4%) | 26 (89.7%) | 1 | 0.38 | 99.3 | 108.8 |
|  |  | G/G | 10 (19.6%) | 3 (10.3%) | 0.52 (0.12-2.31) |  |  |  |
|  | **Overdominant** | A/A-G/G | 30 (58.8%) | 12 (41.4%) | 1 | 0.38 | 99.3 | 108.9 |
|  |  | G/A | 21 (41.2%) | 17 (58.6%) | 1.55 (0.58-4.18) |  |  |  |
|  | **Log-additive** | --- | --- | --- | 0.90 (0.43-1.90) | 0.79 | 100 | 109.6 |
| **rs2073246** | **Codominant** | C/C | 20 (39.2%) | 9 (32.1%) | 1 | 0.8 | 100.8 | 112.6 |
|  |  | C/T | 22 (43.1%) | 16 (57.1%) | 1.24 (0.42-3.68) |  |  |  |
|  |  | T/T | 9 (17.6%) | 3 (10.7%) | 0.75 (0.14-4.02) |  |  |  |
|  | **Dominant** | C/C | 20 (39.2%) | 9 (32.1%) | 1 | 0.82 | 99.2 | 108.6 |
|  |  | C/T-T/T | 31 (60.8%) | 19 (67.9%) | 1.13 (0.39-3.22) |  |  |  |
|  | **Recessive** | C/C-C/T | 42 (82.3%) | 25 (89.3%) | 1 | 0.59 | 98.9 | 108.4 |
|  |  | T/T | 9 (17.6%) | 3 (10.7%) | 0.66 (0.14-3.06) |  |  |  |
|  | **Overdominant** | C/C-T/T | 29 (56.9%) | 12 (42.9%) | 1 | 0.57 | 98.9 | 108.4 |
|  |  | C/T | 22 (43.1%) | 16 (57.1%) | 1.34 (0.49-3.61) |  |  |  |
|  | **Log-additive** | --- | --- | --- | 0.96 (0.45-2.05) | 0.91 | 99.2 | 108.7 |
| **rs2295222** | **Codominant** | C/C | 27 (50.9%) | 11 (39.3%) | 1 | 0.5 | 101.3 | 113.3 |
|  |  | C/A | 22 (41.5%) | 16 (57.1%) | 1.31 (0.47-3.66) |  |  |  |
|  |  | A/A | 4 (7.5%) | 1 (3.6%) | 0.35 (0.03-4.17) |  |  |  |
|  | **Dominant** | C/C | 27 (50.9%) | 11 (39.3%) | 1 | 0.77 | 100.6 | 110.2 |
|  |  | C/A-A/A | 26 (49.1%) | 17 (60.7%) | 1.16 (0.42-3.17) |  |  |  |
|  | **Recessive** | C/C-C/A | 49 (92.5%) | 27 (96.4%) | 1 | 0.29 | 99.6 | 109.1 |
|  |  | A/A | 4 (7.5%) | 1 (3.6%) | 0.30 (0.03-3.33) |  |  |  |
|  | **Overdominant** | C/C-A/A | 31 (58.5%) | 12 (42.9%) | 1 | 0.44 | 100.1 | 109.7 |
|  |  | C/A | 22 (41.5%) | 16 (57.1%) | 1.48 (0.55-3.99) |  |  |  |
|  | **Log-additive** | --- | --- | --- | 0.93 (0.40-2.14) | 0.86 | 100.7 | 110.2 |
| **rs4904155** | **Codominant** | C/C | 18 (35.3%) | 9 (32.1%) | 1 | 0.38 | 98.3 | 110.2 |
|  |  | G/C | 21 (41.2%) | 16 (57.1%) | 1.13 (0.37-3.47) |  |  |  |
|  |  | G/G | 12 (23.5%) | 3 (10.7%) | 0.41 (0.08-2.06) |  |  |  |
|  | **Dominant** | C/C | 18 (35.3%) | 9 (32.1%) | 1 | 0.83 | 98.2 | 107.7 |
|  |  | G/C-G/G | 33 (64.7%) | 19 (67.9%) | 0.89 (0.30-2.60) |  |  |  |
|  | **Recessive** | C/C-G/C | 39 (76.5%) | 25 (89.3%) | 1 | 0.17 | 96.4 | 105.9 |
|  |  | G/G | 12 (23.5%) | 3 (10.7%) | 0.38 (0.09-1.62) |  |  |  |
|  | **Overdominant** | C/C-G/G | 30 (58.8%) | 12 (42.9%) | 1 | 0.41 | 97.6 | 107.1 |
|  |  | G/C | 21 (41.2%) | 16 (57.1%) | 1.52 (0.56-4.14) |  |  |  |
|  | **Log-additive** | --- | --- | --- | 0.72 (0.34-1.50) | 0.37 | 97.5 | 106.9 |
| **rs4904210** | **Codominant** | G/G | 18 (36%) | 7 (25%) | 1 | 0.45 | 98.6 | 110.4 |
|  |  | G/C | 22 (44%) | 18 (64.3%) | 1.63 (0.51-5.18) |  |  |  |
|  |  | C/C | 10 (20%) | 3 (10.7%) | 0.70 (0.13-3.73) |  |  |  |
|  | **Dominant** | G/G | 18 (36%) | 7 (25%) | 1 | 0.57 | 97.9 | 107.4 |
|  |  | G/C-C/C | 32 (64%) | 21 (75%) | 1.38 (0.45-4.21) |  |  |  |
|  | **Recessive** | G/G-G/C | 40 (80%) | 25 (89.3%) | 1 | 0.34 | 97.4 | 106.8 |
|  |  | C/C | 10 (20%) | 3 (10.7%) | 0.50 (0.11-2.20) |  |  |  |
|  | **Overdominant** | G/G-C/C | 28 (56%) | 10 (35.7%) | 1 | 0.23 | 96.8 | 106.3 |
|  |  | G/C | 22 (44%) | 18 (64.3%) | 1.85 (0.67-5.11) |  |  |  |
|  | **Log-additive** | --- | --- | --- | 0.95 (0.44-2.05) | 0.9 | 98.2 | 107.7 |

a. OR: Odds Ratio (Confidence Interval).

b. AIC: Akaike’s Information Criteria.

c. BIC: Bayesian Information Criteria.
