# Supplementary material for: Family and case–control genetic study of MSX1 polymorphisms in peg-shaped teeth Jordanian population
Source: BMC Oral Health. 2022 Jan 22;22:16. doi: 10.1186/s12903-022-02051-2 (PMC8783454; doi:10.1186/s12903-022-02051-2)
Supplement: Supplementary file 2 — Additional file 2. Table S2: Genetic association analysis of all six SNPs polymorphisms in peg-shaped tooth cases and controls using different genetic models. [file 12903_2022_2051_MOESM2_ESM.docx]

**Table S2.** Genetic association analysis of all six SNPs polymorphisms in Peg-shaped teeth cases and controls using different genetic models.

| SNP ID | Category Test | Odds Ratio | 95% CI^a^ | *Chi-square** |
| --- | --- | --- | --- | --- |
| rs12532 | Het (GA) vs Common Hz (AA) | 0.92 | 0.41-2.07 | 0.04 |
|  | Rare Hz (GG) vs Het (GA) | 0.75 | 0.20-2.78 | 0.08 |
|  | Rare Hz (GG) vs Common Hz (AA) | 0.69 | 0.20-2.43 | 0.34 |
| rs1907998 | Het vs (AG) Common Hz (AA) | 0.53 | 0.22-1.24 | 2.18 |
|  | Rare Hz (GG) vs Het (AG) | 1.00 | 0.34-2.93 | 0.00 |
|  | Rare Hz (GG) vs Common Hz (AA) | 0.53 | 0.17-1.61 | 1.27 |
| rs3821949 | Het (AG) vs Common Hz (GG) | 0.57 | 0.26-1.26 | 2.00 |
|  | Rare Hz (AA) vs Het (AG) | 2.95 | 0.25-34.85 | 0.80 |
|  | Rare Hz (AA) vs Common Hz (GG) | 1.69 | 0.14-19.64 | 0.18 |
| rs4464513 | Het (GT) vs Common Hz (GG) | 1.12 | 0.49-2.54 | 0.07 |
|  | Rare Hz (TT) vs Het (GT) | 1.24 | 0.39-3.93 | 0.13 |
|  | Rare Hz (TT) vs Common Hz (GG) | 1.38 | 0.42-4.51 | 0.29 |
| rs6446693 | Het (CT) vs Common Hz (TT) | 1.36 | 0.56-3.30 | 0.46 |
|  | Rare Hz (CC) vs Het (CT) | 0.66 | 0.23-1.87 | 0.62 |
|  | Rare Hz (CC) vs Common Hz (TT) | 0.90 | 0.28-2.88 | 0.03 |
| rs8670 | Het (CT) vs Common Hz (CC) | 1.50 | 0.68-3.31 | 1.01 |
|  | Rare Hz (TT) vs Het (CT) | 0.48 | 0.10-2.26 | 0.89 |
|  | Rare Hz (TT) vs Common Hz (CC) | 0.72 | 0.16-3.31 | 0.18 |

a. CI: Confidence Interval.

*For significant association *P-*value should be < 0.05 and According to the test if *Chi-squared* >3.84 the *P*-value would be < 0.05. None of the SNPs were significant.
