# Supplementary figures and images for: Family and case–control genetic study of MSX1 polymorphisms in peg-shaped teeth Jordanian population
Source: BMC Oral Health. 2022 Jan 22;22:16. doi: 10.1186/s12903-022-02051-2 (PMC8783454; doi:10.1186/s12903-022-02051-2)

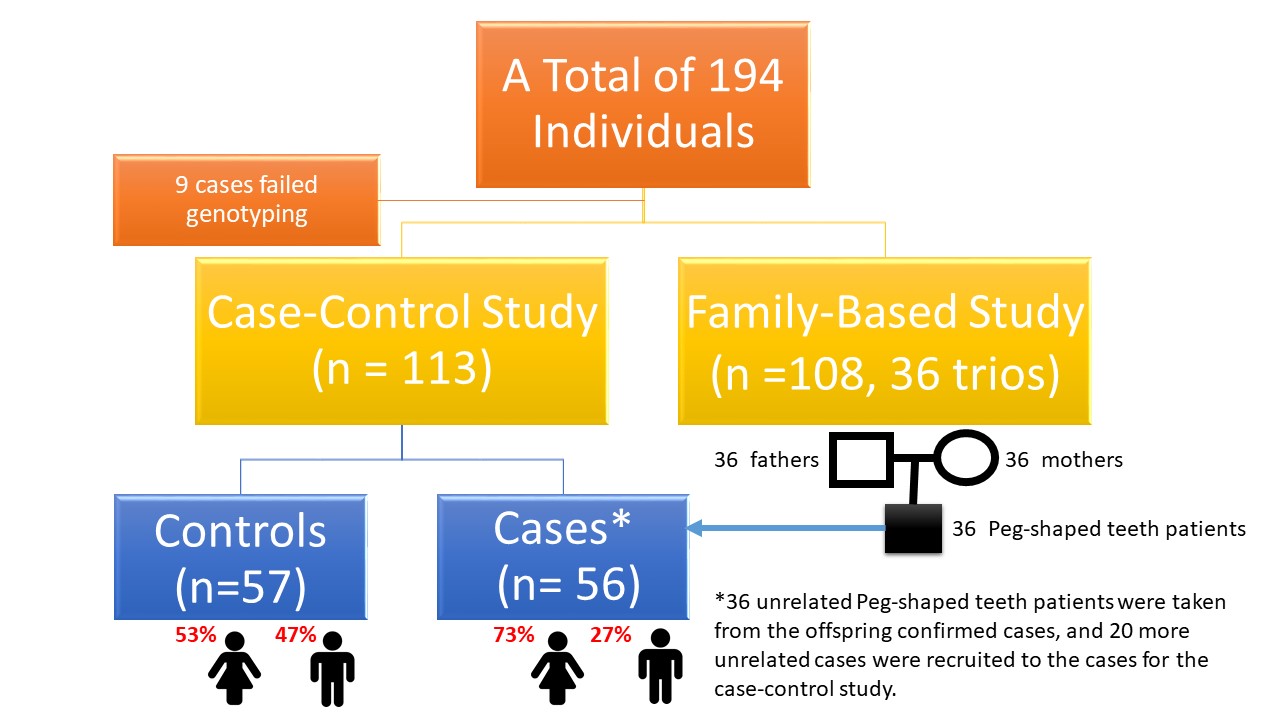

Supplement: Supplementary file 3 — Additional file 3. Fig. S1: Genetic association analysis of all six SNPs polymorphisms in peg-shaped tooth cases and controls using different genetic models. [file 12903_2022_2051_MOESM3_ESM.jpg]
